# Research Priorities in the Mechanical Diagnosis and Therapy Among Diploma Therapists: An International Delphi Study

**DOI:** 10.7759/cureus.62492

**Published:** 2024-06-16

**Authors:** Hiroki Chiba, Yusuke Handa, Kazuki Kikkawa, Hiroshi Takasaki

**Affiliations:** 1 Health and Social Services, Saitama Prefectural University, Koshigaya, JPN; 2 Physical Therapy, Saitama Prefectural University, Koshigaya, JPN

**Keywords:** mckenzie method, musculoskeletal, research priority, physical therapy, delphi method

## Abstract

Introduction

Mechanical diagnosis and therapy (MDT) is the most researched approach in musculoskeletal physical therapy and involves classifying patients into sub-groups based on their response to loading strategies. MDT diploma therapists (Dip-MDTs) fully recognize the value of MDT in musculoskeletal physical therapy. MDT is updating its system based on the latest research, but the system has not yet been fully established. Therefore, more research is required to increase the comprehensibility of the system. Thus, we aimed to identify future research priorities of MDT.

Methods

We conducted a modified Delphi study with three rounds. The Research Advisory Committee (RAC) members (n=7) of the McKenzie Institute International were invited to participate in the initial idea generation round. In the subsequent two consensus rounds, Dip-MDTs (n=299) were asked to indicate their level of agreement with the results of the idea generation round using a five-point Likert scale. The criteria for consensus were mean score ≥4.0, coefficient of variation ≤30%, percent agreement ≥75%, and quartile deviation ≤1. A post-hoc analysis of the consensus was conducted when the number of participants from a certain country exceeded one-third of the total number of participants.

Results

The participation rates for each round were 57.1%, 52.2%, and 49.8%. The USA accounted for 43.6% and 44.3% of all participants at the two consensus rounds, respectively. Finally, nine items reached consensus in the second consensus round. After the post-hoc analysis, eight items reached consensus: (1) cost-effectiveness, (2) cervical and thoracic spine, (3) extremity classifications, (4) filtered randomized controlled trials (RCTs), (5) spinal source classification, (6) centralization and directional preferences, (7) predictive factors for recurrence, and (8) patient education.

Conclusion

In this study, we identified the research priorities of MDT that would enhance the completion of the MDT system.

## Introduction

The stratified care model has become a trend in musculoskeletal physical therapy in recent years [1]. The model classifies treatment types into three algorithms based on (1) prognostic factors, (2) underlying mechanisms, and (3) symptom and functional response to specific interventions [1]. In the third algorithm, classification reliability is critical and the McKenzie method of mechanical diagnosis and therapy (MDT) has been the most investigated and promising in classification reliability [2,3].

MDT offers education in 40 countries and its educational curriculum is the same all over the world. There are two levels of certification in MDT education: (1) credentialed therapists (Cred-MDTs) with basic competence in MDT theory and practice through 100 hours training and (2) diploma therapists (Dip-MDTs) with the highest level of competence in MDT theory and practice through 640 hours training in addition to Cred-MDTs. Based on the latest research, MDT continually updates its educational curriculum and manuals, where review from experts is conducted. Thus, MDT values research and the McKenzie Institute International (MII) has a Research Advisory Committee (RAC), which promotes research activities. All members of the MII RAC have extensive research experience and hold a Cred-MDTs or higher and a Master's degree or higher.

The effectiveness of MDT for low back pain is different between meta-analyses, and its effectiveness has not yet been established [4,5]. Furthermore, compared with the lumbar spine, there are fewer studies on the cervical and thoracic spine [6,7]. As for the reliability of the classification, it has been established for the lumbar spine in Cred-MDTs and Dip-MDTs, but not yet for the cervical spine or extremities [2,3]. Therefore, the system is not fully established by research and more research is required. Thus, we aimed to identify future research priorities of MDT.

## Materials and methods

Study design

The Delphi method is commonly used for identifying a consensus of research priorities as it offers advantages such as (1) limited bias due to no face-to-face discussions, guaranteeing participant anonymity, and (2) the possibility of surveying many geographically diverse participants cost-effectively [8]. However, there is a concern for the quality of the Delphi method because conclusions can be affected by the quality of participants [8]. In MDT, since MDT RAC members are publicly listed and Dip-MDTs are certified by examination, targeting these populations would address the concerns of the Delphi method mentioned above.

In the classic Delphi method, a panel of experts specializing in a particular area engages in iterative rounds until a consensus is reached. However, due to expected challenges such as data aggregation for Dip-MDTs worldwide and decreasing response rates with subsequent rounds, resulting in reduced generalizability, we implemented two modifications in this study. First, an idea generation round was conducted separately, followed by two consensus rounds. Second, the number of rounds was predetermined to be three, including the idea generation round, considered the most frequently used round in Delphi studies [9]. The data were collected for the MII members in the idea generation round and for the Dip-MDTs in the consensus rounds.

Ethical approval for this study was obtained from the Research Ethics Committee of the Saitama Prefectural University (No. 20522), and participants provided their consent by responding to the survey. This study was designed and reported following the Delphi method guidelines, the Conducting, and Reporting of Delphi Studies [10].

Participants

The Delphi method does not clearly specify the number of participants [8]. However, the more participants there are, the better the generalizability [8]. Therefore, in order to include as many participants as possible, the inclusion criteria for the idea generation round were all MII RAC members (n=7), and the inclusion criteria for the first and second consensus rounds were all global Dip-MDTs registered on March 27, 2021 (n=299). The exclusion criterion was those who had missing responses.

Data collection

Data were collected on the web using SurveyMonkey (SurveyMonkey.com, CA, USA). Each round of data collection lasted for one month, with reminders sent every other week. The MII RAC members were directly contacted through email to request study participation in the idea generation round. We requested the MII to send our invitation letter to registered Dip-MDTs in the first and second consensus rounds.

In the idea generation round, participants were asked to propose future research priorities for MDT in an open-ended format. Subsequently, content analysis was conducted by four authors with diverse backgrounds, clinical experience (9.0±6.7 years), MDT qualifications (two have obtained Cred-MDTs and two are in training for the Cred-MDT), and physical therapy-related degrees (one with a doctorate degree, one with a master's degree, and two with bachelor's degrees).

Item grouping was conducted anonymously, and the categories were named independently by the authors [8]. This process was conducted simultaneously until consensus was reached among all authors and a list of research priorities was developed.

In the first consensus round, participants were asked to rate their agreement with the list of MDT research priorities utilizing the commonly used five-point Likert scale (strongly disagree = 1; disagree = 2; neither agree nor disagree = 3; agree = 4; strongly agree = 5) [11]. The proportions of responses for each response category were calculated and included in a new list of research priorities.

In the second consensus round, participants were again asked to rate their agreement with the MDT research priorities on the newly developed list using the five-point Likert scale [11].

For secondary outcomes, demographic information such as gender, age, years of experience as a physical therapist, and time since obtaining the Cred-MDT were collected in the idea generation round. In the first and second consensus rounds, participants’ gender, age, years of experience as a physical therapist, time since obtaining the Dip-MDT, physical therapy-related degree, and nationality were collected.

Data analysis

Descriptive statistics were calculated using IBM SPSS Statistics for Windows, Version 21.0 (Armonk, NY: IBM Corp). In this study, the consensus criteria were more stringent than the general criteria, and they met all of the following criteria: (1) a mean score of ≥4.0, (2) a coefficient of variation of ≤30%, (3) percentage of score 4 or 5 (percent agreement) ≥75%, and (4) a quartile deviation ≤1 [8,9,11,12]. A post hoc analysis of the consensus was conducted when the number of participants from a certain country exceeded one-third of the total number. The research priorities that achieved consensus were categorized for further discussion by two authors (HC and HT).

## Results

The participation ratios for the idea generation, first, and second consensus rounds were 57.1% (n=4, four countries), 52.2% (n=156, 25 countries), and 49.8% (n=149, 23 countries), respectively (Table 1).

**Table 1:** Characteristics of participants in each round of the Delphi study

Variables	Round 1 (n=156)	Round 2 (n=149)
Gender (male)	101 (64.7%)	95 (67.8%)
Age (year)	47.1±10.4	47.0±10.2
Years since acquisition of physical therapy license (year)	22.6±10.6	22.0±10.4
Years since acquisition of medical doctor license (year)	NA	30
Years since the acquisition of the Dip-MDT (year)	10.6±8.1	10.3±7.9
Degree	-	-
Doctor of philosophy	43 (27.6%)	39 (26.2%)
Research master	5 (3.2%)	6 (4.0%)
Clinical master	34 (21.8%)	28 (18.8%)
Bachelor	63 (40.4%)	64 (43.0%)
Less than the bachelor's degree	11 (7.1%)	12 (8.1%)

In the first and second consensus rounds, participants from the USA accounted for 43.6% and 44.3% of all participants, respectively.

The idea generation round yielded 50 open-ended responses. After content analysis (Table 6 of Appendix), these responses were aggregated into 34 items (Table 7 of Appendix). Overall, 13 items in the first consensus round (Table 2) and nine items in the second consensus round (Table 3) met the consensus criteria. 

**Table 2:** Results of the first consensus round of research priorities *The item has been agreed upon. MDT, mechanical diagnosis and therapy; RCTs, randomized controlled trials

	Research priority	Mean score	Coefficient of variation	Percent agreement	Quartile deviation
1*	Studies regarding the cost-effectiveness of MDT	4.5	18.6	88.5	0.5
2*	Studies regarding the cervical and thoracic spine in general	4.3	18.0	86.5	0.5
3*	Studies regarding MDT classifications for extremity musculoskeletal conditions in general	4.3	20.2	84.0	0.5
4*	Studies regarding predictive factors to prevent recurrence after discharge from MDT	4.2	20.4	83.3	0.5
5*	Studies regarding centralization of the cervical and thoracic spine	4.2	21.7	80.1	0.5
6*	Studies regarding pathophysiologies where the symptoms of the extremities are decreased with loading strategies on the spine	4.2	21.7	84.0	0.5
7*	Studies regarding RCTs that include the MDT classification among the inclusion criteria	4.2	20.3	79.5	0.5
8*	Studies regarding the superiority of MDT over other management systems in preventing recurrence	4.1	26.1	84.0	0.5
9*	Studies regarding effective patient education	4.1	21.7	77.6	0.5
10*	Studies regarding the role of centralization and directional preferences as treatment effect modifiers, including studies on sub-groups in non-centralizer with directional preference classifications and its optimal management	4.1	21.7	78.8	0.5
11*	Studies regarding the significance of a mechanical approach to a chronic pain state	4.0	22.8	78.2	0.5
12*	Studies regarding MDT classification of dysfunction syndrome in extremity problems (e.g., prevalence, long-term outcomes)	4.0	25.0	77.6	0.5
13*	Studies regarding extremity problems in general	4.0	24.2	75.0	0.6
14	Studies regarding the benefits of including MDT training in undergraduate education	4.0	25.8	71.8	1.0
15	Studies regarding behavior modification of patients by MDT	3.9	22.8	75.6	0.5
16	Studies regarding the application of MDT to telerehabilitation	3.9	26.6	65.4	1.0
17	Cohort studies comparing initial responders and non-responders	3.9	22.6	70.5	0.5
18	Studies regarding the effect of MDT on nociplastic pain	3.9	24.3	69.9	1.0
19	Studies regarding the effect of repetitive loading to the spine on neuropathic pain	3.9	23.0	69.9	0.5
20	Research on the correlations of centralizer/non-centralizer with directional preference to functional changes	3.9	23.1	68.6	0.5
21	Studies regarding the appropriate load for dysfunction syndrome	3.9	25.1	67.3	1.0
22	Case studies leading to new areas of research and clinical applications of MDT	3.8	25.9	66.0	1.0
23	Studies regarding the clinical reasoning skills of MDT therapists at different stages of MDT education	3.8	27.5	64.7	1.0
24	Studies regarding patient satisfaction depending on the practitioner’s MDT level	3.8	26.7	65.4	1.0
25	Studies regarding the role of the level of MDT education in terms of reliability between therapists	3.8	25.0	66.7	0.5
26	Studies regarding behavioral changes among therapists before and after MDT education	3.7	25.8	64.1	0.5
27	Studies regarding valid and reliable evaluation methods for the recovery of function	3.7	26.5	59.6	0.5
28	Identifying effective interventions during the recovery of function	3.7	26.0	58.3	0.5
29	Studies regarding patient values for self-management and passive treatment (qualitative study)	3.7	27.4	57.7	0.5
30	Studies regarding the underlying mechanisms of centralization and derangement	3.6	29.4	55.8	1.0
31	Studies regarding the predictive ability of MDT classification for postoperative pain relief	3.5	26.5	51.3	0.5
32	Development of criteria for discharge from MDT management	3.5	28.9	49.4	0.5
33	Studies regarding the effect of MDT on presenteeism	3.4	27.4	37.2	0.5
34	Qualitative research on how therapists perceive their role	3.2	32.4	32.7	0.5

**Table 3:** Results of the second consensus round of research priorities *The item has been agreed upon. MDT, mechanical diagnosis and therapy; RCTs, randomized controlled trials

	Research priority	Mean score	Coefficient of variation	Percent agreement	Quartile deviation
1*	Studies regarding the cost-effectiveness of MDT	4.4	20.5	87.9	0.5
2*	Studies regarding the cervical and thoracic spine in general	4.3	18.7	89.3	0.5
3*	Studies regarding MDT classifications for extremity musculoskeletal conditions in general	4.2	22.0	86.6	0.5
4*	Studies regarding RCTs that include the MDT classification among the inclusion criteria	4.1	22.3	79.9	0.5
5*	Studies regarding pathophysiologies where the symptoms of the extremities are decreased with loading strategies on the spine	4.1	23.0	83.2	0.5
6*	Studies regarding the role of centralization and directional preferences as treatment effect modifiers, including studies on sub-groups in non-centralizer with directional preference classifications and its optimal management	4.1	19.4	81.9	0.5
7*	Studies regarding predictive factors to prevent recurrence after discharge from MDT	4.1	21.7	80.5	0.5
8*	Studies regarding centralization of the cervical and thoracic spine	4.1	22.1	80.5	0.5
9*	Studies regarding effective patient education	4.0	20.7	77.2	0.5
10	Studies regarding the significance of a mechanical approach to a chronic pain state	4.0	23.2	71.1	1.0
11	Studies regarding the superiority of MDT over other management systems in preventing recurrence	3.9	27.6	70.5	1.0
12	Studies regarding the benefits of including MDT training in undergraduate education	3.8	28.9	62.4	1.0
13	Studies regarding behavior modification of patients by MDT	3.8	23.7	71.8	0.5
14	Research on the correlations of centralizer/non-centralizer with directional preference to functional changes	3.8	23.0	66.4	0.5
15	Identifying effective interventions during the recovery of function	3.7	25.6	65.1	0.5
16	Studies regarding the effect of repetitive loading to the spine on neuropathic pain	3.7	24.8	63.1	0.5
17	Studies regarding extremity problems in general	3.7	25.3	65.8	0.5
18	Cohort studies comparing initial responders and non-responders	3.7	23.6	60.4	0.5
19	Studies regarding the clinical reasoning skills of MDT therapists at different stages of MDT education	3.7	26.8	61.1	0.5
20	Studies regarding the effect of MDT on nociplastic pain	3.7	22.5	56.4	0.5
21	Case studies leading to new areas of research and clinical applications of MDT	3.7	27.2	59.7	0.5
22	Studies regarding the underlying mechanisms of centralization and derangement	3.6	29.8	57.0	0.5
23	Studies regarding MDT classification of dysfunction syndrome in extremity problems (e.g., prevalence and long-term outcomes)	3.6	24.8	59.1	0.5
24	Studies regarding valid and reliable evaluation methods for the recovery of function	3.6	27.2	55.7	0.5
25	Studies regarding the application of MDT to telerehabilitation	3.6	26.3	53.7	0.5
26	Studies regarding the role of the level of MDT education in terms of reliability between therapists	3.6	27.3	61.7	0.5
27	Studies regarding patient values for self-management and passive treatment (qualitative study)	3.6	26.1	57.7	0.5
28	Studies regarding behavioral changes among therapists before and after MDT education	3.6	27.8	56.4	0.5
29	Studies regarding the appropriate load for dysfunction syndrome	3.6	26.6	56.4	0.5
30	Studies regarding patient satisfaction depending on the practitioner’s MDT level	3.5	27.6	55.0	0.5
31	Development of criteria for discharge from MDT management	3.4	28.2	47.7	0.5
32	Studies regarding the predictive ability of MDT classification for postoperative pain relief	3.3	30.7	43.0	0.5
33	Studies regarding the effect of MDT on presenteeism	3.2	23.9	29.5	0.5
34	Qualitative research on how therapists perceive their role	3.0	32.2	25.5	0.5

A post-hoc analysis of the consensus was conducted using data from participants in the USA and the rest of the world. The post-hoc analysis showed that of the nine items that met the criteria for consensus in the second consensus round, only "Studies regarding centralization of the cervical and thoracic spine" dropped out. Regardless of whether the analysis considered all participants or focused on those from the USA and the rest of the world, eight items consistently met the consensus criteria. The eight items that met the criteria for consensus were as follows: "Studies regarding the cost-effectiveness of MDT," "Studies regarding the cervical and thoracic spine in general," "Studies regarding MDT classifications for extremity musculoskeletal conditions in general," "Studies regarding RCTs that include the MDT classification among the inclusion criteria," "Studies regarding potential pathophysiologies where the symptoms of the extremities are decreased with loading strategies on the spine," "Studies regarding the role of centralization and directional preferences as treatment effect modifiers, including studies on sub-groups in non-centralizer with directional preference classifications and its optimal management," "Studies regarding predictive factors to prevent recurrence after discharge from MDT," and "Studies regarding effective patient education." The eight research priorities (Table 4) were categorized into three themes: (1) effectiveness/efficacy, (2) classification, and (3) prevention (Table 4).

**Table 4:** Agreed research priorities that were finally agreed upon in the Delphi study *effectiveness/efficacy ^†^classification ^‡^prevention MDT, mechanical diagnosis and therapy; RCTs, randomized controlled trials

Research priority	All participants	USA	Rest of the world
Studies regarding the cost-effectiveness of MDT*	✓	✓	✓
Studies regarding the cervical and thoracic spine in general*	✓	✓	✓
Studies regarding MDT classifications for extremity musculoskeletal conditions in general^†^	✓	✓	✓
Studies regarding RCTs that include the MDT classification among the inclusion criteria^†^	✓	✓	✓
Studies regarding potential pathophysiologies where the symptoms of the extremities are decreased with loading strategies on the spine^†^	✓	✓	✓
Studies regarding the role of centralization and directional preferences as treatment effect modifiers, including studies on sub-groups in non-centralizer with directional preference classifications and its optimal management^†^	✓	✓	✓
Studies regarding predictive factors to prevent recurrence after discharge from MDT^‡^	✓	✓	✓
Studies regarding effective patient education^‡^	✓	✓	✓
Studies regarding centralization of the cervical and thoracic spine	✓	✓	
Studies regarding the superiority of MDT over other management systems in preventing recurrence		✓	

## Discussion

This is the first study to identify future MDT research priorities. Here, we identified eight research priorities using a two-round modified Delphi method.

Synthesis of the eight research priorities

Theme - Effectiveness/Efficacy

The research priorities that reached consensus, specifically “Studies regarding the cost-effectiveness of MDT” and “Studies regarding the cervical and thoracic spine in general,” were categorized under this theme. Currently, studies on cost-effectiveness are limited to LBP [13]. While many randomized controlled trials (RCTs) and meta-analyses have indicated the effectiveness of MDT for LBP, research on the effectiveness of MDT for the cervical and thoracic spines is relatively limited [4,6,7]. Thus, further studies regarding the effectiveness and efficacy of MDT in these regions are required.

Theme - Classification

The MDT uses classifications that guide management strategies rather than relying on pathoanatomical classifications. Based on this, the following research priorities that reached a consensus were categorized under this theme: “Studies regarding MDT classifications for extremity musculoskeletal conditions in general,” “Studies regarding RCTs that include MDT classification among their inclusion criteria,” “Studies regarding potential pathophysiologies where loading strategies on the spine reduce symptoms in the extremities,” and “Studies regarding the role of centralization and directional preferences as treatment effect modifiers, including sub-groups of non-centralizers with directional preference classifications and optimal management strategies.”

RCTs that include MDT classification among their inclusion criteria have recently emerged in the literature, acknowledging the heterogeneity of musculoskeletal pain within certain pathoanatomically diagnostic labels [14,15]. Further validation of the MDT classification is needed to increase the comprehensibility of MDT. In this validation process, factors such as the role of centralization and directional preference as treatment effect modifiers and the spinal source of symptoms in the extremities should be explored [16,17].

Theme - Prevention

The research priorities of “Studies regarding predictive factors to prevent recurrence after discharge from MDT” and “Studies regarding effective patient education” that reached a consensus were categorized under this theme. Musculoskeletal pain is highly recurrent, resulting in significant economic burdens for individuals and society [18-20]. Thus, further studies are necessary to explore effective patient education methods and identify important factors that contribute to preventing musculoskeletal pain recurrence. Previous studies have identified several contributory factors to LBP recurrence and have generally suggested exercise as a useful way to reduce LBP recurrence, regardless of the type [19,21]. However, the recurrence rate of LBP among individuals who have completed MDT treatment remains untested. A previous study comparing a short-term MDT-based self-management education group with a non-intervention group after LBP improvement found no statistically significant difference in LBP recurrence rates at one year but had fewer visits to medical institutions [22]. Such an enhanced resilience to medical service through MDT was also detected in a study of neck pain [23]. As there is preliminary evidence that patients who underwent MDT changed their attitudes toward their own health, it is hypothesized that MDT led patients to change their behavior so that even if symptoms are recurrent, they can manage them independently and avoid additional medical care [24]. However, there has been no direct evidence for the hypothesis and thus we assumed that evidence of MDT in preventing recurrence was required.

Inconsistent priorities in the post-hoc analysis

The research priorities of “Studies on cervical and thoracic spine centralization” and “Studies on the superiority of MDT over other management systems in preventing recurrence” met the consensus criteria among participants from the USA but not those from the rest of the world or all participants. First, for the former priority, clinical practice guidelines strongly recommend centralization-based interventions for LBP, but no guideline recommendation currently exists for the cervical spine in the USA [25,26]. Thus, physical therapists in the USA may seek further verification of the effectiveness of MDT on the cervical and thoracic spine.

For the latter priority, the Movement System Impairment Diagnostic System has been incorporated into the vision of the APTA to prevent musculoskeletal disorder recurrence and flare-ups [27]. Therefore, clinicians practicing MDT in the USA may be interested in comparing MDT to other management systems.

Comparisons to previous studies on research priorities in musculoskeletal disorders

In the previous scoping review reported in 2018, research priorities for musculoskeletal disorders were categorized into 13 areas with a weighting of the number of research priorities reported in previous studies [28]. A comparison of the research priorities of this study and the previous study is summarized in Table 5.

**Table 5:** Comparison of the research priorities of the previous study and this study *it can be applicable to more than one item. “Studies regarding the cervical and thoracic spine in general” and “Studies regarding MDT classifications for extremity musculoskeletal conditions in general” were not considered applicable to any other area.

Bourne et al. (2018)	This study
Epidemiology and burden (4.1%)	No corresponding priority
Etiology and risk factors (6.5%)	Studies regarding potential pathophysiologies where the symptoms of the extremities are decreased with loading strategies on the spine
Screening, diagnosis, and assessment (1.0%)	Studies regarding RCTs that include the MDT classification among the inclusion criteria. Studies regarding the role of centralization and directional preferences as treatment effect modifiers, including studies on sub-groups in non-centralizers with directional preference classifications and its optimal management
Prevention (1.7%)	*Studies regarding predictive factors to prevent recurrence after discharge from MDT. Studies regarding effective patient education
Treatment (39.5%)	No corresponding priority
Natural history, prognosis, and outcome (7.5%)	*Studies regarding predictive factors to prevent recurrence after discharge from MDT
Outcome measurement (3.1%)	No corresponding priority
Economic evaluation (2.7%)	Studies regarding the cost-effectiveness of MDT
Implementation (2.0%)	No corresponding priority
Health services and systems (5.1%)	No corresponding priority
Research capacity building (9.5%)	No corresponding priority
Research methods (4.1%)	No corresponding priority
Patient/consumer perspective (13.9%)	No corresponding priority
	Studies regarding the cervical and thoracic spine in general
	Studies regarding MDT classifications for extremity musculoskeletal conditions in general

While treatment accounted for the largest proportion of research priorities in the previous study, there were no applicable items in this study [28]. On the other hand, "Screening, diagnosis and assessment," which had the smallest percentage in previous studies, accounted for the largest percentage in this study at 40％ (two out of eight items). These results may be related to MDT emphasis on the ADTO model [29]. The ADTO model is a concept that will guide future manual physical therapy research designs and is important in the stratified care model [29]. The acronym ADTO is derived from Assessment-Diagnosis-Treatment-Outcome [29]. The model has three links and recommends that the studies be conducted in the following order: A-D link (establishing intra- and inter-rater reliability of classifications), D-T link (establishing the responsiveness of multiple treatments to a particular classification), and T-O link (determining which of the interventions shown in the previous step is actually the most effective). Although MDT has established classification reliability for the spine, it has not yet established classification reliability for the extremities [2,3]. Therefore, to improve the completeness of the MDT system, we believe that further research at the level of the A-D link is needed first.

Strengths of this study

We used well-defined criteria to identify experts, whereas Delphi studies often suffer from ambiguity in determining the population of experts [8]. In addition, the criteria for consent are more stringent by including more items than in previous studies. Therefore, we believe that the certainty of evidence from the current study is high.

Limitations

A potential limitation of this study may be the use of the Delphi method. First, while the survey population is maintained for each round in the original Delphi method, we used different populations for the idea generation and consensus survey rounds. However, this limitation would not compromise the value of this study, as the RAC members completed the clinical training diploma in MDT, and their viewpoints reflect those of the Dip-MDTs. The number of consensus survey rounds was set to two based on previous studies, as no clear guidelines exist on the optimal number of rounds [9]. Although further selection could be achieved by increasing the number of rounds, we do not believe that specifically selecting priorities would enhance the value of the study, as a certain consensus has already been reached.

The second limitation is that the number of participants decreased as the rounds progressed. In the Delphi method, which is an anonymous survey, the participation rate commonly decreases as the round progresses [8]. Some believe that the Delphi method should have more than 70% participation in each round, but there is no clear appropriate definition [8]. The 70％ criterion has not been met in previous physical therapy studies [30]. On the other hand, the participation rate in the third round of this study was also higher than in the previous study [30]. Therefore, we believe that the study limitations were not sufficient to affect the validity of the results of this study.

Another limitation of the study is the large number of participants from the USA, which might have influenced the global results. However, we do not consider this a limitation that compromises the value of the study, as the post-hoc analysis revealed that eight of the nine research priorities overlapped between the USA and the rest of the world.

## Conclusions

In this study, we identified future research priorities in Dip-MDTs worldwide. Research priorities on eight of the nine were agreed upon by all regions. The eight research priorities could be categorized into three themes: effectiveness/efficacy, classification, and prevention. Addressing these priorities is expected to advance the completion of the MDT system.

## Appendices

**Table 6:** Comparison table between comments in the first round and results in content analysis MDT, mechanical diagnosis and therapy; RCTs, randomized controlled trials

Comments in the first round		Results in content analysis
Many more studies in all areas of Mechanical Diagnosis and Therapy (MDT) classifications in extremity problems	1	Studies regarding MDT classifications for extremity musculoskeletal conditions in general
MDT classification in extremities versus traditional orthopedic diagnoses - prevalence and management value		
In general more regarding extremity problems, all areas	2	Studies regarding extremity problems in general
In general more regarding thoracic spine	3	Studies regarding the cervical and thoracic spine in general
In general more regarding cervical spine		
Dysfunction in extremity problems - % and long-term outcomes	4	Studies regarding MDT classification of dysfunction syndrome in extremity problems (e.g., prevalence, long-term outcomes)
Dysfunction in extremity problems versus non-dysfunction - % and long-term outcomes		
Equivalent positive responders in the extremity problems - % and long-term		
Dysfunction classification is very under-researched. May not feature with high prevalence in spinal problems? But definitely very important in extremity problems.		
Equivalent of centralization/directional preference in thoracic spine - % and long-term	5	Studies regarding centralization of the cervical and thoracic spine
Centralization/directional preference in the cervical spine; especially proportion (%) and long-term studies (one year)		
Patient satisfaction of patients receiving MDT versus non-MDT	6	Studies regarding patient satisfaction depending on the practitioner’s MDT level
Studies regarding underlying mechanisms where the symptoms of the extremities are decreased with loading strategies on the spine	7	Studies regarding potential pathophysiologies where the symptoms of the extremities are decreased with loading strategies on the spine
Why does "education" work/not work; what are the successful elements; what are the most important components of "education"?	8	Studies regarding effective patient education
Studies regarding the effect of repetitive loading to the spine on neuropathic pain	9	Studies regarding the effect of repetitive loading to the spine on neuropathic pain
Studies regarding the effect of MDT on nociplastic pain	10	Studies regarding the effect of MDT on nociplastic pain
What therapists think that their role is in treating and managing patients (qualitative study)	11	Qualitative research on how therapists perceive their role
What sub-groups in non-centralizer/directional preference?	12	Studies regarding the role of centralization and directional preferences as treatment effect modifiers, including studies on sub-groups in non-centralizer with directional preference classifications and its optimal management
How best to manage non-centralizer/directional preference?		
Role of centralization/directional preference as treatment effect modifiers - what is the best treatment?		
Clinical reasoning of MDT versus non-MDT therapists	13	Studies regarding the clinical reasoning skills of MDT therapists at different stages of MDT education
Clinical reasoning of MDT therapists at different stages of MDT education		
Role of the level of MDT education in reliability between therapists	14	Studies regarding the role of the level of MDT education in terms of reliability between therapists
Development of criteria for discharge from MDT management	15	Development of criteria for discharge from MDT management
What are the cost savings of using the MDT approach compared with the "standard approach" to back pain, neck pain, shoulder pain, and knee pain?	16	Studies regarding the cost-effectiveness of MDT
Studies regarding the cost-effectiveness of MDT		
All comers, traditional RCTs versus filtered RCTs, in which some selection/management after classification occurs, as in Long et al. (2004)	17	Studies regarding RCTs that include the MDT classification among the inclusion criteria
What is the physiological process that causes the centralization of symptoms and concomitant improvement in the affected range of motion?	18	Studies regarding the underlying mechanisms of centralization and derangement
Studies regarding the underlying mechanisms of derangement		
Studies regarding the underlying mechanisms of centralization		
Correlate centralization/directional preference classification with functional changes	19	Research on the correlations of centralizer/non-centralizer with directional preference to functional changes
The limited value of case studies needs to be recognized; although these are useful to explore new areas of MDT, as preliminary studies, there is a need for more rigorous and generalizable studies, some of which are outlined below reliability of assessment procedures in all areas - spinal and extremity	20	Case studies leading to new areas of research and clinical applications of MDT
Studies regarding behavioral changes among therapists before and after MDT education	21	Studies regarding behavioral changes among therapists before and after MDT education
Studies regarding the predictive ability of MDT classification for postoperative pain relief	22	Studies regarding the predictive ability of MDT classification for postoperative pain relief
Studies regarding behavior modification of patients by MDT	23	Studies regarding behavior modification of patients by MDT
Establish why it is important to teach entry-level students the MDT approach before entering practice	24	Studies regarding the benefits of including MDT training in undergraduate education
Prospective cohort studies - initial responders versus non-responders	25	Cohort studies comparing initial responders and non-responders
What proportion of initial responders and non-responders change classification, and what happens to them?		
Studies regarding the superiority of MDT over other management systems in preventing recurrence	26	Studies regarding the superiority of MDT over other management systems in preventing recurrence
Does MDT really reduce the occurrence or/and the impact of future episodes		
Studies regarding predictive factors to prevent recurrence after discharge from MDT	27	Studies regarding predictive factors to prevent recurrence after discharge from MDT
Studies regarding the appropriate load for dysfunction syndrome	28	Studies regarding the appropriate load for dysfunction syndrome
More patients' voices (qualitative studies)	29	Studies regarding patient values for self-management and passive treatment (qualitative study)
What do patients think about passive versus active management strategies?		
What do patients think about "self-management" versus treatment done by the therapist "to" them?		
Identifying effective interventions during the recovery of function	30	Identifying effective interventions during the recovery of function
Studies regarding valid and reliable evaluation methods for the recovery of function	31	Studies regarding valid and reliable evaluation methods for the recovery of function
Studies regarding the significance of a mechanical approach to a chronic pain state	32	Studies regarding the significance of a mechanical approach to a chronic pain state
Studies regarding the application of MDT to telerehabilitation	33	Studies regarding the application of MDT to telerehabilitation
Studies regarding the effect of MDT on presenteeism	34	Studies regarding the effect of MDT on presenteeism

**Table 7:** Results of the content analysis MDT, mechanical diagnosis and therapy; RCTs, randomized controlled trials

Research priority	
1	Studies regarding MDT classifications for extremity musculoskeletal conditions in general
2	Studies regarding extremity problems in general
3	Studies regarding the cervical and thoracic spine in general
4	Studies regarding MDT classification of dysfunction syndrome in extremity problems (e.g., prevalence and long-term outcomes)
5	Studies regarding centralization of the cervical and thoracic spine
6	Studies regarding patient satisfaction depending on the practitioner’s MDT level
7	Studies regarding potential pathophysiologies where the symptoms of the extremities are decreased with loading strategies on the spine
8	Studies regarding effective patient education
9	Studies regarding the effect of repetitive loading to the spine on neuropathic pain
10	Studies regarding the effect of MDT on nociplastic pain
11	Qualitative research on how therapists perceive their role
12	Studies regarding the role of centralization and directional preferences as treatment effect modifiers, including studies on sub-groups in non-centralizer with directional preference classifications and its optimal management
13	Studies regarding the clinical reasoning skills of MDT therapists at different stages of MDT education
14	Studies regarding the role of the level of MDT education in terms of reliability between therapists
15	Development of criteria for discharge from MDT management
16	Studies regarding the cost-effectiveness of MDT
17	Studies regarding RCTs that include the MDT classification among the inclusion criteria
18	Studies regarding the underlying mechanisms of centralization and derangement
19	Research on the correlations of centralizer/non-centralizer with directional preference to functional changes
20	Case studies leading to new areas of research and clinical applications of MDT
21	Studies regarding behavioral changes among therapists before and after MDT education
22	Studies regarding the predictive ability of MDT classification for postoperative pain relief
23	Studies regarding behavior modification of patients by MDT
24	Studies regarding the benefits of including MDT training in undergraduate education
25	Cohort studies comparing initial responders and non-responders
26	Studies regarding the superiority of MDT over other management systems in preventing recurrence
27	Studies regarding predictive factors to prevent recurrence after discharge from MDT
28	Studies regarding the appropriate load for dysfunction syndrome
29	Studies regarding patient values for self-management and passive treatment (qualitative study)
30	Identifying effective interventions during the recovery of function
31	Studies regarding valid and reliable evaluation methods for the recovery of function
32	Studies regarding the significance of a mechanical approach to a chronic pain state
33	Studies regarding the application of MDT to telerehabilitation
34	Studies regarding the effect of MDT on presenteeism
